# A systematic review of explanatory factors of barriers and facilitators to improving asthma management in South Asian children

**DOI:** 10.1186/1471-2458-14-403

**Published:** 2014-04-27

**Authors:** Monica Lakhanpaul, Deborah Bird, Logan Manikam, Lorraine Culley, Gill Perkins, Nicky Hudson, Joanne Wilson, Mark Johnson

**Affiliations:** 1 General and Adolescent Paediatrics Unit, UCL Institute of Child Health, 30 Guilford Street, London WC1N 1EH, UK; 2 Chelsea and Westminster NHS Foundation Trust, 369 Fulham Road, London SW10 9NH, UK; 3 Department of Primary Care and Public Health Sciences, 5th Floor, Capital House, 42 Weston Street, Guy’s, London SE1 3QD, UK; 4 School of Applied Social Sciences, De Montfort University, The Gateway, Leicester LE1 9BH, UK; 5 Canterbury Christ Church University, North Holmes Road, Canterbury Kent CT1 1QU, UK; 6 Leicester Children’s Community Services, Bridge Park Plaza, Bridge Park Road, Thurmaston, Leicester LE4 8PQ, UK; 7 Mary Seacole Research Centre, De Montfort University, The Gateway, Leicester LE1 9BH, UK

**Keywords:** Asthma, Barriers, Facilitators, Asians

## Abstract

**Background:**

South Asian children with asthma are less likely to receive prescriptions and more likely to suffer uncontrolled symptoms and acute asthma admissions compared with White British children. Understanding barriers are therefore vital in addressing health inequalities. We undertook a systematic review identifying explanatory factors for barriers and facilitators to asthma management in South Asian children. South Asians were defined as individuals of Indian, Pakistani or Bangladeshi descent.

**Methods:**

Data Sources - Medline, HMIC, EMBASE, ASSIA, Web of Science, BNI, CINAHL, PsycINFO, OpenSIGLE, CRD, Scopus, NHS Evidence, Cochrane Library, Campbell Collaboration, RCPCH, ATS, ERS, Asthma UK, Google Scholar & Asthma Guidelines (BTS, GINA, ATS, Monash, NAEPP, Singapore & New Zealand) to August 2013.

Inclusion Criteria – Qualitative, quantitative or mixed methods research with primary focus on identifying explanations for barriers and/or facilitators to asthma management in South Asian children aged 0–18 years with diagnosed/suspected asthma and/or carers and/or healthcare professionals.

Data Extraction – Three authors independently reviewed, selected & extracted eligible articles with disagreements resolved by research team discussion.

**Results:**

15 studies encompassing 25,755 children, 18,483 parents/carers and 239 healthcare professionals were included. Barriers and explanatory factors identified were:

1. Lack of asthma knowledge in families and healthcare professionals.

2. Under-use of preventer medications.

3. Non-acceptance/denial of asthma.

4. Over-reliance on Emergency Department management.

5. Communication problems.

6. Non-adherence to medication.

7. Use of complementary therapies.

Little facilitators regarding asthma management were identified.

**Conclusions:**

Several key issues were identified as likely to be ethnic-specific to South Asian families, rather than a reflection of minority status: impact of parental and professional knowledge and beliefs, health service utilisation pattern explanations and the impact of prejudice and stigmatisation. Other explanations such as language barriers are not strictly ethnic specific but instead reflect a minority position.

Further research is required to identify why barriers exist, the mechanisms by which they impact on asthma management and how they can be overcome. Furthermore, understanding the difference between barriers and explanations that are ethnic-specific and those that are related to being a minority will enable the application of generic system-wide interventions where ethnicity is not the issue and ethnically-tailored interventions where needed.

## Background

Asthma is a common chronic condition responsible for significant childhood morbidity with a substantial care burden on families, communities and health services [1]. A 13-trial meta-analysis [2], noted that South Asian children diagnosed with asthma were less likely to receive prescriptions for reliever and preventer medications compared to White British children. Further UK data demonstrate that South Asian children with asthma are more likely to suffer uncontrolled symptoms and be admitted to hospital with acute asthma with no evidence to suggest that they have more severe asthma [2-4].

Seid and Sobo [5,6] posited that ‘barriers to care’, particularly in vulnerable and minority communities, impact on outcomes. Understanding and systematically addressing barriers are therefore vital in addressing health inequalities [6,7]. Whilst barriers to asthma management amongst minority ethnic groups are well documented, little has been done to understand why these barriers exist or to identify how they might be addressed. Similarly, there is scant evidence published on identifying and explaining facilitators to asthma management in minority ethnic community groups.

We therefore undertook a systematic review of primary evidence exploring explanations of barriers and facilitators to asthma management in South Asians. We aimed to identify whether explanations relate specifically to ethnicity or to being in a minority and therefore sought evidence from South Asian settings where research was designed around ‘local’ cultural norms and ‘South Asian’ origin population groups.

This review intends to inform community-based intervention development as an alternative to imposition of health promotion approaches tried and tested only on the majority population, which have failed to prevent inequalities in minority groups [8,9]. In this review, ‘South Asian’ refers to individuals of Indian, Pakistani or Bangladeshi descent with non-modifiable factors (genetic variations and asthma phenotypes) excluded.

## Methods

### Search question

The PICO and SPICE acronyms are established models for aiding systematic searches [10]. SPICE was used to delineate all elements of our research question.

**S**etting: general practice, hospital, primary care, community care

**P**erspective: minority ethnic community children with asthma and/or parents of children with asthma

**I**ntervention: management processes

**C**omparison: asthma control

**E**valuation: barriers/facilitators

### Eligibility criteria

The original aim was to source all articles including primary research, reviews, abstracts, conference proceedings and unpublished studies with a focus on minority ethnic community children in any country or South Asian children in minority or majority settings. Due to the vast literature, only full text primary research was included in the final analysis.

Studies were included if they met the following criteria:

• Setting: Intention to study South Asian children/families in either minority or majority settings

• Participants: South Asian children aged 0–18 yrs with diagnosed or suspected asthma and/or their carers and/or their healthcare professionals

• Intentions: Primary focus on identifying explanations for barriers and/or facilitators to asthma management

• Design: Qualitative, quantitative or mixed

### Information sources

An information scientist with specialist interest in ethnicity research derived the search strategy and searched the following databases and web resources supported by JW, LM and DB: MEDLINE, HMIC, EMBASE, ASSIA, Web of Science – SSCI and SCI, BNI, CINAHL, PsycINFO, OpenSIGLE, HTA, DARE, Scopus, Social Care Online, NHS Evidence, The Cochrane Library, Campbell Collaboration; CRD York, NICE, British Thoracic Society; American Thoracic Society; European Thoracic Society; Asthma UK. 5636 citations were returned.

Bibliographies of relevant articles, asthma management guidelines and previous systematic review were also hand searched by DB, LM and JW: 3735 citations were searched. Databases were searched for evidence from 1990 onwards with English-language translations available. Initial searches were conducted in 2009 and updated in May 2010.

### Definitions

Asthma management: relates to a process of treatment with the aim of achieving and maintaining asthma control.

Barriers: factors that impede the achievement and maintenance of asthma control and that include (but are not limited to) health beliefs, professional barriers, language and communication, prejudice or discrimination, delivery and organisation issues, issues around diagnosis, and treatment offered.

Facilitator: the inverse of a barrier (factors that assist achievement or maintenance of control).

South Asian: those of Indian, Pakistani or Bangladeshi descent.

Children: persons less than 18 years of age.

### Search strategy

The search strategy included terms for “asthma”, “South Asian” (including terms specifying all major subgroups), and “children”. For example, the search strings used for Medline were:

• Asthma OR wheez* AND

• Ethni* OR minorit* OR rac* OR South Asian OR Indian* OR Pakista* OR Banglades* OR urban OR inner-city AND

• Child* OR young OR adolescen* OR pediatri* OR paediatri* OR parent* OR mother* OR father* OR carer*

### Study selection and data extraction

**Figure 1:**
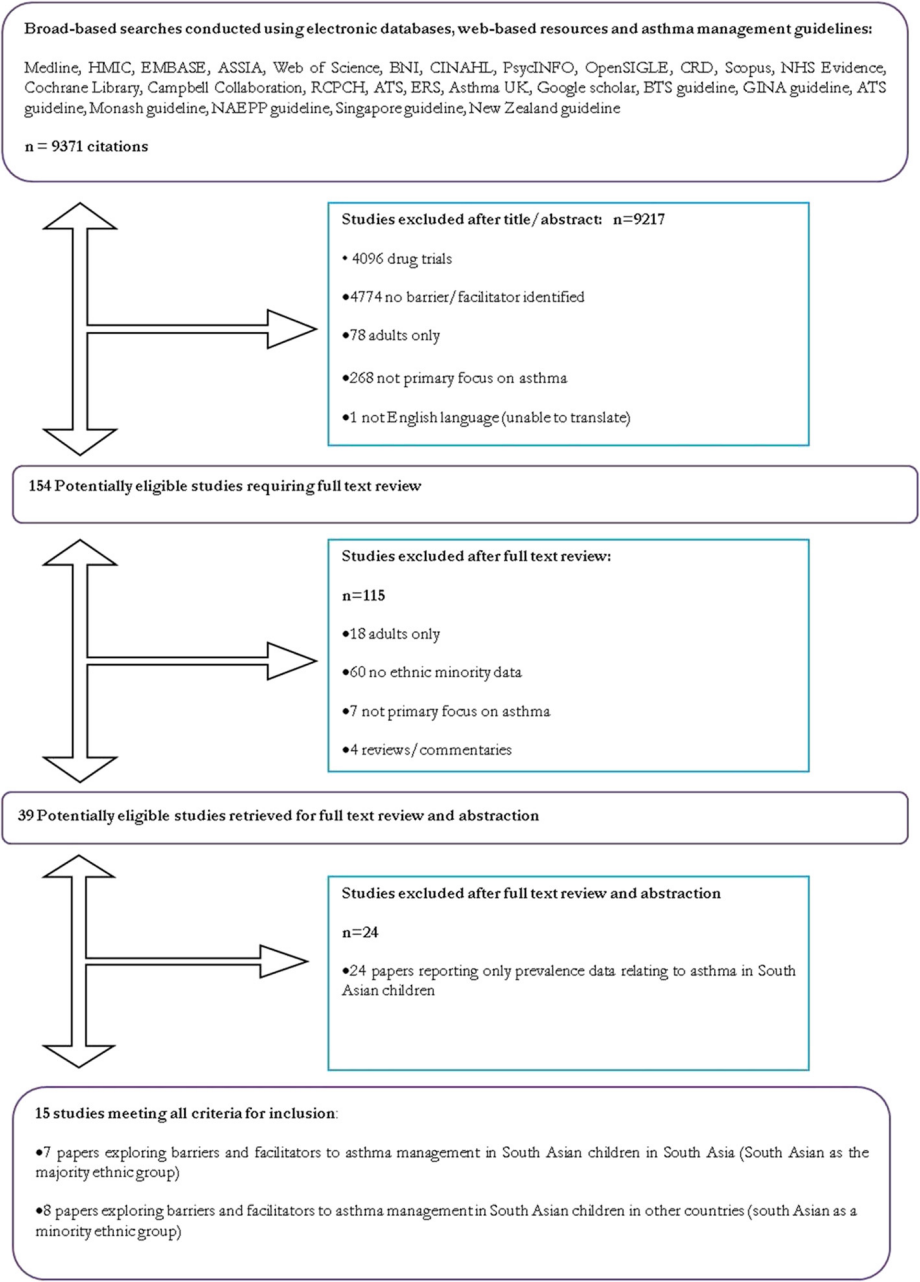
Study selection flow diagram.

GP, DB, LM and JW screened 9371 titles and abstracts against inclusion criteria: 9217 were excluded at this stage. A further 154 full papers were retrieved and scan-read where eligibility could not be determined by abstract alone: 108 papers were excluded at this stage. This left 46 potentially eligible articles for full text review and data extraction. Two reviewers assessed these papers independently, conflicts being resolved by discussion with the team. The study selection process is denoted in Figure 1.

GP extracted data using a piloted modified CASP worksheet [11] including: country of study; study aims; population studied, eligibility criteria and illness diagnosis; study design; ethical approval; sampling; data collection and analysis; presentation of the results; discussion of the findings; bias; value of the research; summary critique and limitations. DB conducted a reduced data extraction for all potentially eligible studies, and a full data extraction for 20% of the potentially relevant studies, with 95% agreement between GP and DB.

### Quality assurance

The Centre for Reviews and Dissemination (CRD) guidance emphasises the importance of using a structured approach to quality assessment when assessing qualitative studies for inclusion in reviews. However, it acknowledges the lack of consensus on the definition of poor quality [12]. Some argue that using rigid quality criteria may lead to the unnecessary exclusion of papers [13], and suggest that instead of excluding papers on quality grounds, a critique be included in the narrative to allow the reader to decide. This is the method used in this review.

### Result synthesis

The eligible studies tended to address very broad research questions, were conducted using qualitative and/or quantitative methods and were not presented in the format of controlled trials. The evidence reviewed is presented as a narrative report together with a critical reflection of the synthesis process used to assess its robustness [13-15].

## Results

We included 15 studies with a primary focus on explaining barriers or facilitators to asthma management in South Asian children.

### Study and participant characteristics

15 studies comprising of 25,755 children aged between 2 and 17 years (age range not specified in one paper), 18,483 parents/carers of children and 239 healthcare professionals working with children were included. 14 studies were quantitative (13 involving questionnaires) and 1 was qualitative. Outcomes measured included knowledge/understandings/beliefs about asthma (7 studies), symptom perception/understandings of ‘wheeze’ (3 studies), prescriptions and use of preventer medications (2 studies), impact of migration and ‘westernisation’ on asthma and atopy (2 studies). Two studies took place in Pakistan, five in India (South Asian majority) and eight in the UK (South Asian as minority).

For ease of reading the data have been grouped into themes, corresponding to a barrier or facilitator with evidence for any explanatory factors. These are denoted in Table 1.

**Table 1:** Table of included studies

**Study (country)**	**Study type**	**Population**	**Barriers**	**Facilitators**	**Critique**
Kuehni^3^ (UK)	Prevalence survey	6080 children aged 1-4	Possible under-treatment with steroids	NS	High response rate
Hazir^15^ (Pakistan)	Questionnaire based interview	200 parents/carers of children with asthma aged 2–13; attended hospital asthma clinic between 3 m-7 y	Lack of understanding of medication use, food beliefs, social stigma & poor child self-esteem	Lack of awareness not significantly related to socioeconomic or educational background. Community strategies to raise awareness needed.	Pakistan is an ethically, culturally & socially diverse country. Hospital based study therefore may not reflect true situation in community.
Shivbalan^16^ (India)	Questionnaire survey	100 children aged 2–15 with total >4 wheeze episodes, 2 wheeze episodes in the last 6 months with at least 2 ED visits and 1 hospitalisation.	Lack of knowledge and acceptance about asthma, poor understanding of aetiology & prognosis, misconceptions about long-term medications, social stigma & reliance on GPs for information	Awareness of triggers	No clear details on ethical approval or eligible/recruited numbers. Majority of participants from same socioeconomic status therefore may not be representative
Haque^17^ (Pakistan)	Questionnaire survey pre/post seminar	82 GPs registered with the College of Family Medicine	Lack of knowledge by healthcare professionals	NS	Participants were GPs who voluntarily attended an educational programme & therefore results may be biased towards motivated GPs
Gautam^18^ (India)	Questionnaire survey	157 GPs registered with the Delhi Medical Association	Knowledge gaps in different GPs. Includes diagnosis, misconceptions about food and exercise avoidance and parental smoking effects	NS	No clear inclusion/exclusion criteria & mention questionnaire validity. Non-respondent bias may be present–43 (21.5%) GPs refused.
Lai^19^ (India)	Questionnaire survey	85 children with asthma ages 6–17 with minimum 2 years since symptom onset.	Poor physician-parent communication, social stigma, misconceptions about food avoidance & beliefs that modern medicines cause harm	Parents keen to learn & parental recognition of importance of treating asthma	No clear recruitment methodology & mention of questionnaire validity. Participants enrolled in asthma clinic so biased towards those receiving medical care.
Ormerod^20^ (UK)	Prevalence survey	1783 adults and children with asthma aged 0–70 registered with participating GP practice	Asthma under-diagnosis with possible under-recognition & reporting	NS	No clear recruitment methodology and no sample size calculations. Findings reflect Blackburn GPs so may not be generalisable.
Duran-Tauleria^21^ (UK)	Questionnaire survey	14490 children aged 5–11 with respiratory symptoms including asthma, wheeze & bronchitis66	NS	Ethnic monitoring and targets for specific populations to monitor adherence to clinical guidelines & indicators to monitor inequalities in asthma treatment in minority ethnic communities	No clear sampling & recruitment methodology & no clear inclusion/exclusion criteria.
Cane^22^ (UK)	Focus groups	66 mothers aged 22–45 from Bangladeshi, White or Black Caribbean backgrounds.	Different (sometimes inaccurate) understandings of asthma, use of alternative medications, delay in seeking Western medical help & stigma	NS	Study based on mothers’ perception of video of child with an asthma attack with lack of further content. Unclear analysis methodology. No data on socioeconomic or educational background collected.
Smeeton^23^ (UK)	Questionnaire survey	150 parents of children with asthma aged 3-9	Stigma, erroneous beliefs & choosing not to give medications	NS	Clear recruitment and sampling methodology with clear analysis. High proportion of SA participants born outside UK with low education level & therefore may impact results.
Singh^24^ (India)	Questionnaire survey	1012 adults and children with asthma	Lack of knowledge about asthma, failure of recognising warning symptoms, beliefs in permanent cure, use of complementary medicine & treatment non-adherence	Children preferred inhalers whereas adults preferred oral medications	No data on questionnaire validity. No clear eligibility, inclusion & exclusion criteria. Use of numerous closed questions. Study and analysis included both adults and children.
Mittal^25^ (India)	Questionnaire survey	52 child–parent pairs; children aged 6–15 diagnosed with asthma	Parent and child ability to perceive symptom severity (influenced by child’s age), cigarette smoke exposure and asthma severity	NS	Unclear reason of chosen sampling and recruitment method.
Michel^26^ (UK)	Questionnaire survey	4236 children aged 6-10	English as second language & deprivation	Higher maternal education.	Parents received three study questionnaires so may have had a learning effect. Low response rates of 52% of Whites & 40% of South Asians.
Panico^27^ (UK)	Cohort study	14630 singleton infants aged 3 whose mothers participated in the survey	Language & maternal migration – suggests the lack of UK familiarity & language skills leads to underreporting of asthma	NS	Despite large study size small SA group samples (5%). Barriers are inferred. Children of mixed ethnicity classified according to the EM parent’s group and may lead to effect attenuation.
Carey^28^ (UK)	Prevalence survey	847 children aged 8–11 with asthma, atopy or bronchial hyperreactivity	Western diet associated with more hyperreactivity	Asian diet appears protective	No data on questionnaire reliability and validity.

NS = none specified.

### Information and knowledge about asthma

None of the included studies specifically explored knowledge with UK resident South Asian families. Two studies did this in South Asian families resident in Pakistani and India [16,17]. Both identify that parental knowledge was lacking but neither paper explored the reasons for this.

Poor knowledge about asthma was identified amongst family physicians in Pakistan [18], [50% of physicians failed a 17 item questionnaire and 52% failed on management approach]. Physicians in India fared better with >75% of clinicians correctly answering questions on treatment importance, preventative medications use and steroid safety [19], however this study was biased as physicians could opt out of questionnaire completion. In both studies, newer graduates scored better than older graduates, suggesting that failure to refresh knowledge amongst older professionals may be explanatory. Assuming physicians as the primary information source for patients and families it is possible that poor physician knowledge translates to poor family knowledge [20].

### Under-use of preventative medications

Treatment non-compliance is a frequent explanation for poor outcomes in minority ethnic communities. The evidence however, supports two alternative explanations: under-diagnosis of asthma and under-prescription of preventer medications. One UK based prevalence study reported significant discrepancies between the percentage of children with diagnosed asthma and those with symptoms of asthma (labelled probable asthma) [21]. In contrast, another UK based prevalence study noted that inhaled corticosteroid [ICS] prescriptions were lower in South Asian compared to White children [OR 0.46, CI 0.28-0.77 in 1-year-olds, and 0.63, CI 0.44-0.91 in 2–4-year-olds], despite no evidence for asthma severity variation [3].

Finally, in a large UK based quantitative survey it was noted that Indian Subcontinent children with acute asthma were less likely to use the correct administration method or receive medications (OR 0.35, CI 0.22-0.57), compared to White and Other inner-city children (OR 0.82, CI 0.49-1.38) [22]. This suggests that under-prescription in South Asian children is related to the South Asian ethnicity and not to being a minority group. Whilst it appears unlikely that such under-treatment in the UK is related to medicine unavailability, exploration of such associations in both UK and South Asia in the literature remain sparse [3,22].

### Non-acceptance/denial of diagnosis

Two studies in India and Pakistan reported that 48% and 61% of parents respectively did not accept their child’s asthma [17,20]. Stigmatisation may be explanatory. In two studies based in India and another in Pakistan it was noted between 26% and 37% of parents believed asthma to be contagious [16,17,20]. In one, 50% of parents hesitated disclosing to others about their child’s asthma [20]. It is suggested that these findings reflect asthma associated social stigma impacting on treatment compliance.

This is reflected in a UK based focus group study which noted Bangladeshi participants suggesting that ‘other’ Bangladeshi people view asthma as contagious and that asthma associated stigma was present in Bangladesh but not in UK [23]. This was also noted in another UK study where Indian and Pakistani parents were the most likely to believe that their child did not have asthma compared to White parents [OR 4.04, CI 1.04-15.76] [24]. Although there appears to be an ethnicity-specific belief that asthma is contagious and stigmatising, the prevalence and impact of such beliefs may be lessening amongst minority (i.e. diasporic) South Asian population as a result of acculturation.

### Reliance on Emergency Department [ED] services in ‘acute’ attacks

Several explanatory factors were identified for over-reliance on ED care. One may be inaccurate symptom assessment or underestimation of asthma severity. In an India-based questionnaire survey of Indians, the majority of patients were unable to assess their symptom severity with only 11.9% able to perceive warning signs of an acute attack [25]. A similar study undertaken in India compared the accuracy of parents and their children in recognising asthma severity symptoms and noted 62% and 60% accuracy respectively [26]. When peak flow measurements were performed, 92% of parents and 89% of children had underestimated asthma severity, most noticeably amongst those with severe asthma compared to mild/moderate disease [26].

Another factor may be beliefs concerning appropriate place of treatment. A UK-based study noted that South Asian parents were noted to more likely believe that ‘better help and support’ was available via hospitals rather than their GP [OR 4.27, CI 1.67 to 10.96] [24]. This was in contrast to another study of Bangladeshi mothers in the UK that noted that whilst GPs are recognised as the first step to treatment they would go to ED if their GP was inaccessible [23].

### Communication barriers

Communication encompasses many elements, one of which is language. Two papers support the existence of a language barrier in South Asian children asthma management. In a questionnaire study to identify parental understanding of wheeze in UK White and South Asian parents, 83.5% correctly identified wheeze as a whistling or squeaking noise. A correct answer however, was less likely if parents did not speak English as a first language [10]. This study however was hampered by low response rates and the administration of 3 study questionnaires within 5 years that may have led to ‘correct’ answer learning.

In another UK-based cohort study examining understandings of asthma and wheeze a large proportion of Bangladeshi mothers were noted to need help with translated questionnaires and suggested that language barriers may interfere with symptom reporting ability [27]. With the absence of language barriers being reported in data from South Asia we can conclude that language barriers reflect being in a minority position rather than an ethnicity-specific issue, although clearly language barriers may exist with specific ethnic groups within India, and language may be the defining feature of ethnic-difference that is associated with minority status among people of migrant origin in Europe and elsewhere.

### Non-adherence to medication

Treatment non-adherence is a well-known barrier to asthma management. In the UK, it was reported that South Asian parents were less likely to give regular inhaled corticosteroids (ICS), more likely than White parents to believe that medicines were addictive [OR 3.89, CI 1.47-10.27] and that medicine does more harm than good [OR 3.19, CI 1.22-8.34] [24]. Similar results were noted in South India that reported that amongst 100 children requiring acute asthma treatment (i.e. nebulisers) at least once, only 12 children used inhalers at home. Additionally, parents reported that aerosol therapy was addictive and continuous medication use during symptom free intervals would impair the child’s ability to grow out of asthma with 47% of parents not aware that inhalers could be administered at home [17]. This supports the notion that knowledge and beliefs are closely linked and that misconceptions around medication side effects may underlie non-adherence.

Medication format may also be an explanatory factor. 82% of Pakistani parents in Pakistan felt that inhalers were superior to oral treatment, despite 77% currently using oral treatments [16]. In contrast, a questionnaire study of parental attitudes towards western and complementary medicine in India noted only 43% of adult and children were prescribed inhalers and only 48% of children chose to use inhalers over oral medications [25]. It suggests that reluctance to use inhalers relates to stigmatisation, with unproven examples of girls in particular wanting to hide that they had asthma as it may impact upon their marriage prospects.

Complementary medicine is widely practiced in South Asian countries. In a Pakistan-based study, Hazir et al. [16] noted that whilst 98% of Pakistani parents preferred going to the doctor for treatment, 9% also visited a Hakim [traditional practitioner] and 11% a homeopath. Lai et al. [20] reported that 65% of Indian parents sought treatment outside the hospital whilst Singh et al. [25] noted that 79% of Indian parents used alternative therapies to manage their child’s asthma, including ayurvedic treatments, homeopathy and yoga. Finally, in a UK based study, it was reported that Bangladeshi mothers held generally positive attitudes towards the medical profession and western medicines whilst generally accepting western medications as means of controlling asthma symptoms [23].

Whilst complementary therapy use is sometimes suggested as a barrier to asthma management or an explanation for non-adherence to western medications it is not clear whether alternative remedies are used in conjunction with western treatments, or instead of them. Nor is it clear why complementary therapies are used. Though easy to presume that such remedies are traditional, it is possible that parents turn to complementary therapies to fill perceived gaps in western medications. From the evidence, data that links barriers to explanatory factors and management behaviours is lacking.

### Dietary modification

It was noted in a UK based study that whilst White British mothers were concerned about preservatives and food allergies and Black Caribbean mothers advocated junk food avoidance and the importance of a healthy diet, Bangladeshi mothers identified specific foods to be avoided by a child with asthma [bananas, cold milk, ice-cream] and commented that “doctors here don’t even mention restricting foods”. Additionally, specific foods and drinks were reported as avoidable triggers in 89% of parents in a Pakistani study [16], 68% of parents in an Indian study [17] and an unspecified percentage in a second Indian study [20].

Furthermore, 40% of GPs in Delhi [19] believed that drinking milk increased mucus production in asthmatic children, and that asthmatic children should not eat bananas [64%], chilled food [24%], dairy products [53%] sour food [35%], nor drink chilled liquids [41%]. These beliefs were closely correlated to length of time in practice, with more experienced (i.e. older) GPs being more likely to hold these beliefs than newer GPs. Thus dietary modification, over and above healthy eating or food allergy avoidance, appears to be a factor specific to South Asian families, not a reflection of minority status.

### Facilitators to asthma management in south Asian children

Very little was identified relating to facilitators to achieving asthma control in South Asian children. South Asian families appear to have a highly positive attitude towards healthcare professionals: Smeeton et al. [24] found that Indian and Pakistani parents were the most likely to accept/believe that their child had asthma [OR 4.04, CI 1.04-15.76] and Lai et al. [20] reported that Indian parents of asthmatic children viewed their doctors as the major and most reliable source of information. As the prevailing attitude amongst South Asians is to have a strong degree of trust in medical professionals, this should be sensitively explored and utilised to assist families in gaining control over asthma.

Diet may also be protective. In a UK based prevalence survey, Carey et al. [28] reported that for South Asian children, a traditional Asian diet was inversely associated to airway hyper-reactivity risk in a dose response fashion: ORs for hyperreactivity with an exclusively Asian, mostly Asian, or a mixed diet relative to an English diet were 0.31 [95% CI 0.15 to 0.62], 0.88 [0.56 to 1.37], and 0.99 [0.65 to 1.49]. This suggests that South Asian dietary habits may be protective with changing diets associated with second/third generation families and acculturalisation potentially accounting for some variation in asthma outcomes.

## Discussion

This review highlights the importance of both establishing the barriers and exploring why those barriers exist. For each barrier identified, there are various documented and theorised explanations, yet without clearly identifying these factors, overcoming such barriers is difficult.

Several key issues were identified as ethnic-specific to South Asians, rather than simply a minority status reflection: impact of parental and professional knowledge and beliefs, health service utilisation pattern explanations, and impact of prejudice and stigmatisation in the UK. Whilst language barriers reflect a minority position and are not strictly ethnic-specific, this presents a significant challenge due to the impracticality of translating all health service materials and consultations into all UK spoken minority languages. Nevertheless, greater efforts could be made to ensure effective communication with those with lower proficiency in English by the use of booked interpreters for asthma consultations and the use of telephone interpreting services such as Language Line.

### Gaps in the research

A lack of explanations underlying reported barriers was identified. Where offered, they tend to be theorised rather than proven. This reflects the nature of quantitative research that is inherently limited by specific questioning. Whilst qualitative research can be time consuming and costly, particularly if bi-lingual facilitators are used, it remains the best method of identifying underlying explanations for barriers.

Stigmatisation prevalence and its impact on asthma management in South Asians remain poorly understood in the UK, particularly in multi-generation families, extended families and in the context of easy international travel and communication.

The impact of diet on asthma remains underexplored despite its relevance to the South Asian community, given the high prevalence of beliefs on dietary restrictions in children with asthma and suggestions that the South Asian diet is protective against developing asthma.

Healthcare professional bias also requires further investigation. In a review of impact of ethnicity on asthma management [29], it is suggested that subconscious bias and stereotyping affects care quality. Ethnographic research involving direct observation and event questioning as they unfold is likely to be needed to compare and contrast clinical management and expectations in children of different ethnic backgrounds.

Additionally, impact of out-dated professional knowledge and behaviours on patient knowledge and behaviours requires further research. This was identified almost 30 years ago when UK Asian women had antenatal care provided by GPs with insufficient obstetric knowledge [30] and suffered significantly increased perinatal mortality and morbidity. It is suggested that British South Asian families gravitate towards South Asian GPs for both cultural and linguistic reasons. Given the evidence reported in this review, they may be lacking in current asthma knowledge. It can therefore be argued that asthma knowledge amongst GPs should be explored.

### Operational challenges

Whilst we found several studies investigating ethnic differences in prevalence of asthma and medication use, no clear association between a barrier, explanation and management behaviour was identified. Without linkage, there is considerable risk of presuming explanations or impacts on management that do not apply. This has implications for resource use efficiency: tailoring interventions to minority ethnic communities has been shown to be effective in asthma management [31]. It would be inappropriate however, to tailor interventions to all ethnic groups if barriers are not ethnic specific, but rather reflect being a minority group.

Defining ethnicity is challenging. The problem lies in how ethnicity is defined – geographically, linguistically, and historically or another way. In UK studies, various methods were used in defining ‘origin’; mother’s self-identification as Indian, Pakistani or Bangladeshi [3,27,32], 2001 UK census category self-selection [25,28], ethnic group classification by spoken home language, fieldworkers subjective assessment of ethnicity [23], no definition beyond ‘Asian patients’ [22], or no description of ethnicity definition [24]. Using pre-set categories by country of origin is simple, but takes no account of ethnic (linguistic or religious/cultural) variations within countries; and challenging for older generations who may identify with one country but were born in another.

UK census category use allows for generalisation and ease of ethnic group analysis, but is limited by fixed categories changeable with each census. Language use is a pragmatic solution, particularly when work is conducted in multiple languages. Conversely, subjective assessment by a fieldworker is fraught with potential pitfalls. It is argued that allowing ethnicity self-definition without pre-set categories is most effective however this may present significant challenges in analysis and applicability [33]. We join others in recommending that ethnicity be clearly explained in all ethnic health variation research by specifying the way in which ethnicity has been defined and operationalised, so that readers can determine applicability to their own populations [34].

### Application to clinical practice

Given the large number of barriers and explanations that may impact upon asthma management, clinicians may be daunted by the prospect of addressing these barriers in everyday practice. We do not suggest that clinicians ask for an exhaustive social history nor possess an encyclopaedic knowledge of all religions and cultures. Rather, clinicians should take a holistic view which will allow them to see children as existing within their family, social and cultural setting. This encompasses cultural competency; awareness and discussion of barriers and ethnic-specific explanations that exist and their impact on their patient’s management and self-management

Our findings highlight the importance of cultural and community beliefs. For example, dietary restriction is not considered in the widely used British Thoracic Society [BTS] guidelines on asthma management, yet beliefs and practices concerning food and asthma are prevalent amongst UK South Asian families and the possible stigmatisation of asthma in South Asian communities may also present a barrier to timely presentation and diagnosis. At the same time, a lack of culturally competent practice and adequate communication support in the NHS may provide significant system level barriers to effective asthma management in South Asian children.

## Conclusions

Identifying ethnicity-specific barriers is important in developing ethnically tailored intervention programmes. However, the danger in this typology is to overemphasis the importance of ethnicity (and the risk of stereotyping) in influencing asthma management outcomes in minority ethnic community groups. Research should therefore shift towards focussing on clearly linking ethnic specific beliefs, barriers and management practices to clinical outcomes. By understanding this pathway in a variety of settings and ethnic groups, we can then understand the relationships between them. This will allow identification of whether ethnicity or minority group position, and ‘patient’/user or clinician/provider knowledge, is the key factor in management and intervention design.

It can be argued that rather than individuals holding views and beliefs specific to their (minority) ethnicity, these views are not the UK norm within the NHS, which was primarily designed as a ‘one size fits all’ service, not designed to accommodate diverse opinions. For example, services and resources to address food avoidance amongst South Asians in the management of asthma are limited.

Recent NHS structural reforms have has brought sweeping changes across the NHS together with opportunities to re-design services. Diversity could be embraced with a wider range of services commissioned under the NHS umbrella with examples including commissioning and placing alternative or complementary practitioners under the same scrutiny and evidence-based quality assurance process as existing healthcare providers.

In an ideal case, the NHS and its resources as well as the training (initial formative training and continuing professional development) would be redesigned on an assumption of serving a diversity of users with multiple faith, cultural or religious and knowledge systems. Alternatively the NHS could establish itself as a set of core ‘generic’ services, designed to suit the majority population, and with provision of ‘ethnic-specific’ tailored specialist services for identified local groups of minority ethnic backgrounds.

Whilst minimising resource implications, this would in essence create a secondary market and risk suspicion that diversity was being met at the expense of equality or quality. Unless the paradox is addressed, failure to address the increasingly complex and ‘multi-diverse’ populations of the UK will firmly establish that being a member of a minority will adversely impact on the availability, utility and relevance of NHS services and health outcomes.

Provision of ethnic-specific services (and language support) where numbers justify it locally, and greater awareness of diverse explanations and needs, will help reduce health inequalities and reduce the risks associated with under-treatment or mis-management.

## Competing interests

All authors declare that we have no competing interests.

## Authors’ contributions

ML, LC, MJ and DB conceived and participated in the design of the study. DB, LM, NH, GP and JW coordinated and undertook the review. All authors performed the data interpretation and contributed equally to write the draft, read and approve the final manuscript

## Pre-publication history

The pre-publication history for this paper can be accessed here:

http://www.biomedcentral.com/1471-2458/14/403/prepub
